# Assessment of Ultraviolet-C Inactivation of *Pseudomonas aeruginosa* and *Aspergillus flavus* in the Anesthesia Workspace

**DOI:** 10.1016/j.mayocpiqo.2026.100740

**Published:** 2026-07-28

**Authors:** Randy W. Loftus, Franklin Dexter, Michelle C. Parra, Carmen T. Brindeiro, Chase P. Loftus, Cornelie CL. Banguid, Brendan T. Wanta, Jonathan E. Charnin

**Affiliations:** a Department of Anesthesiology and Perioperative Medicine, Mayo Clinic, Rochester, MN; b Department of Anesthesia, University of Iowa, IA; c RDB Bioinformatics, Coralville, IA

## Abstract

**Objective:**

To determine the ultraviolet-C (UV-C) irradiation doses required to inactivate *Pseudomonas aeruginosa* and *Aspergillus flavus* contamination on polycarbonate surfaces using a triangular emitter configuration.

**Patients and Methods:**

In this laboratory experiment, polycarbonate test carriers inoculated with >10^8^ colony-forming units of *P aeruginosa* (MYA-15442) or *A flavus* (American Type Culture Collection 9643) were positioned 2.74 m from 3 linear UV-C emitters to determine minimally effective doses. Ten independent irradiation doses were evaluated for *P aeruginosa* (25-250 mJ/cm^2^) and *A flavus* (50-2000 mJ/cm^2^). Larger carriers were additionally evaluated for *A flavus* to model reduced cell clumping, simulating prior surface disinfection. Residual colony-forming units were quantified and compared with untreated controls to calculate log reductions. Finally, the efficacy of delivering these minimally effective doses through a triangular UV-C emitter configuration was assessed.

**Results:**

For *P aeruginosa*, no growth was detected at any dose, including 25 mJ/cm^2^. For *A flavus* on standard carriers, inactivation occurred at 725 mJ/cm^2^, though no growth conditions were not achieved at any tested dose. However, on larger carriers (reduced clumping), no growth conditions for *A flavus* were achieved at 650 mJ/cm^2^. Delivery of 25 mJ/cm^2^ for *P aeruginosa* and 650 mJ/cm^2^ for *A flavus* through the triangular configuration resulted in undetectable growth.

**Conclusion:**

A UV-C dose of 25 mJ/cm^2^ is sufficient to attenuate *P aeruginosa* surface contamination. Conversely, *A flavus* inactivation will likely require manual high-level surface disinfection augmented with 650 mJ/cm^2^ of UV-C irradiation.

Surgical site infections (SSIs) increase patient morbidity and mortality^1^ and affect up to 11% of patients undergoing surgery.^2^ Pathogen transmission among anesthesia workspace reservoirs (eg, movement from the anesthesia machine to the patient’s nose during induction of anesthesia) is tightly associated with SSI development. The risk of SSI is 2% without transmission and 18% with the transmission of pathogens that are resistant to the prophylactic antibiotic employed for surgery.^3^ Improved cleaning of the anesthesia machine and equipment is a key component of a multifaceted approach that can reduce pathogen transmission and SSIs.^4^, ^5^, ^6^

Anesthesia machines can be disinfected fully with manual cleaning,^7^ but in routine practice there is less than 50% efficacy.^8^^,^^9^ Ultraviolet-C (UV-C) irradiation can augment surface disinfection cleaning of the anesthesia machine, where the impact on major pathogens such as *Staphylococcus aureus* (*S aureus*),^10^ the more pathogenic *S aureus* multilocus sequence type 5 (multilocus sequence type [MLST] 5),^11^ *Clostridioides difficile*,^12^ and *Candida auris*^10^ has been described. The UV-C irradiation delivery approach involved a triangular UV-C emitter configuration, one emitter at the foot and one on each side of the surgical bed.^10^, ^11^, ^12^ This was more effective than single emitters rotating 360° given the ability to combat barriers to irradiation dose delivery such as height, distance, and surface orientation that can result in shadowing.^10^ With the triangular configuration, the minimally effective dose required for achieving no growth conditions for surfaces contaminated with *S aureus* MLST 5 was 27± 0.15 mJ/cm^2^ even without use of chemicals.^11^^,^^13^

Gram-negative pathogens are an additional consideration because they account for a proportion of SSIs,^14^ are implicated in intraoperative transmission events,^15^ and are associated with increased odds of infection when isolated from one or more anesthesia workspace reservoirs such as the anesthesia machine.^16^ *P aeruginosa* is particularly important because it is both routinely isolated from surgical wounds and from the anesthesia machine.^14^, ^15^, ^16^ Other Gram-negative or Gram-variable pathogens that are frequently isolated from surgical wounds such as *Escherichia coli*, *Citrobacter freundii,* and *Bacillus cereus* are infrequently isolated from the anesthesia machine or other anesthesia workspace reservoirs.^14^, ^15^, ^16^ Additional information pertaining to an evidence-based approach for UV-C augmentation of routine cleaning procedures to address *P aeruginosa* is needed.

*Aspergillus flavus* (*A flavus*) is a UV-resistant, opportunistic fungal pathogen that can cause severe infections in immunosuppressed patients. The mode of transmission is often aerosolized, but environmental fomites have been described.^17^ This pathogen can grow on plastic surfaces^18^ and on organic material.^19^ While 99 mJ/cm^2^ has been reported to inactivate (achieve a 2-log reduction) *A flavus*,^20^ the supporting studies involved inactivation in water, which requires a lower dose due to lack of mycelial structure formation and cellular aggregation.^20^ The required dose for inactivation of *A flavus* on polycarbonate surfaces, a common material in the anesthesia workspace,^10^, ^11^, ^12^ is unknown. Given that inactivation times have been reported to be four times longer for polycarbonate material than for stainless steel, it is expected that inactivation of *A flavus* on polycarbonate surfaces will require >99 mJ/cm^2^.^21^ Further understanding of the dose required for inactivation of *A flavus* on polycarbonate surfaces will help to guide UV-C augmentation of surface disinfection cleaning in high-risk clinical environments such as operating room theaters and post-transplant care units that can support *A flavus* growth.^17^

In this study, our primary aim was to determine whether 25 mJ/cm^2^ of UV-C irradiation is effective in attenuating *P aeruginosa* contamination of polycarbonate material.^10^^,^^11^^,^^13^ We also evaluated the irradiation dose required for *A flavus* attenuation.

## Patients And Methods

Because this laboratory experiment (conducted May 8, 2023 to June 15, 2023) did not involve human or animal subjects, tissues, or samples, it was exempt from local ethics committee approval; therefore, no protocol number was assigned.

### Pathogen Carriers and Preparation

Commercially available polycarbonate carriers (ReadyNowTM Test Carriers, Stratix Labs Corporation) were obtained for *P aeruginosa* ATCC MYA-15442 and *A flavus* ATCC 9643.^22^, ^23^, ^24^ Standard polycarbonate slides 1” x 0.9” were utilized for both pathogens. In addition, larger slides of 1” × 3” were used for *A flavus* to explore the potential impact of cell clumping on energy requirements.^25^ Inoculum preparations for pathogen carriers were > 8 log (CFU)/mL to ensure a > 6 log CFU/carrier after plating and drying. The top of the cartridge was removable to allow exposure of the preinoculated carrier to disinfection agents, and the back side of the cartridge had an adhesive strip for mounting the device to vertical test surfaces. These carriers were used for both treatment and control conditions, and all carriers were prepared, managed, and processed for CFU enumeration using identical procedures as outlined below. Additional information pertaining to test material preparation by the manufacturer can be found in the Supplemental Appendix S1, available online at http://www.mcpiqojournal.org.

### Procedures for Control and Treatment Pathogen Carrier UV-C Exposures

A linear configuration of 3 UV-C emitters was used to determine the minimally effective dose for pathogen attenuation (inactivation defined by a ≥2 log reduction (LR) or resulting in no growth). Subsequently, the impact of delivery of the minimally effective dose by the previously studied triangular configuration of emitters^10^, ^11^, ^12^, ^13^ on pathogen attenuation was assessed.

Following previously published methodology,^10^^,^^12^ the laboratory testing area was confirmed to be free from line-of-sight obstacles or obstructions.

Determination of the minimally effective dose for pathogen attenuation: Three UV-C emitters using low-pressure mercury lamps (Surfacide) were positioned in a row in front of an aluminum stand placed at nine feet (2.74 m) from the emitters. The distance to the center of the stand was measured bythe use of a calibrated tape measure from the blue power inlet on the center of the middle emitter. The distance of the emitters reflected coverage of a typical patient room that would also be applicable to the anesthesia workspace.^26^

**Figure 1:**
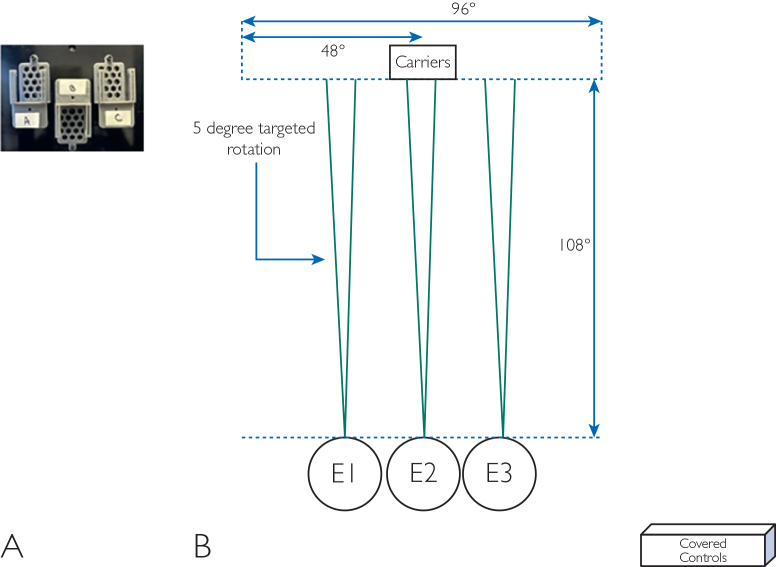
Linear configuration of emitters each rotating 5°. (A) Pathogen carriers were mounted on an aluminum stand (fixture) at three positions (A=center of stand, B=left of center of stand, and C=right of center of stand) and at a height of 47.5 inches from the floor, center of bulbs. The carriers were positioned on the metal plate (upper left corner of the figure) that was attached to the stand by a screw (hole above position B) that allowed consistency of carrier positioning in relation to the center of the stand while adjusting the height and between experiments. (B) The stand was positioned at 9 feet from three low-pressure mercury gas UV-C emitters that were positioned in a row and each rotating 5°. The test carriers and adjacent radiometers were exposed to an increasing dose of UV-C energy. Log reductions (LR) were calculated by comparing the average of treatment samples (final colony-forming units [CFU]) for a given dose to the average of positive controls (initial CFU), log10(initial CFU/final CFU). Following treatment at each target dose, carrier and control slides were removed from the cartridge and processed identically to enumerate CFU. Log reductions were calculated by comparing the average of treatment samples (final CFU) for a given dose to the average of positive controls (initial CFU), log10(initial CFU/final CFU). ∗This image was created by the authors and adapted from © Copyright 2024 Loftus et al,^10^ an open access article distributed under the terms of the Creative Commons Attribution License CC-BY 4.0., which permits unrestricted use, distribution, and reproduction in any medium, provided the original author and source are credited.

Using standard 1” × 0.9” carriers, 10 irradiation doses for *P aeruginosa* (25, 50, 75, 100, 125, 150, 175, 200, 225, and 250 mJ/cm^2^) and *A flavus* (50, 100, 150, 200, 250, 300, 725, 1,150, 1,575, and 2000 mJ/cm^2^) were evaluated independently for each of 3 positions on the stand (left of center [A], center [B], and right of center [C]) and for each of 2 dilutions (10^-6^ and 10^-7^), N=60 assessments for each pathogen (3 positions × 2 dilutions × 10 irradiation doses). Three positive control pathogen carriers open to ambient conditions were present during the entire treatment period for each pathogen and dose combination, shielded from irradiation by placement in a box covered by a tin pan (13 × 21 × 3 in) linxed with aluminum foil (Figure 1). There were in conjunction three negative controls for each pathogen/dose combination. . Using large, 1” × 3” carriers, the above was repeated for *A flavus* at a dose ranging from 50 to 750 mJ/cm^2^.

For each trial, treatment pathogen carriers and radiometers calibrated for the peak germicidal spectrum of 254 nm^27^ (International Light Technologies model 1270) were mounted on the stand at 3 positions (left of center [A], center [B], and right of center [C]), facing and positioned directly ahead of the center emitter at center of lamp (47.5 in from the floor). To measure the delivered irradiation dose, the radiometers were positioned vertically to the emitters. As the intensity of UV-C irradiation varies over the length of the bulbs, with the highest irradiance generated from the center of the bulbs, carriers and radiometers were placed at the center of the bulbs, measured at 47.5 in (120.65 cm) from the floor (Figure 1). The calibrated radiometers measured the band of wavelengths surrounding the peak intensity wavelength. Irradiance, W/cm^2^, or power/cm^2^, delivered to the carriers at a given distance was measured by the radiometers, where the dose delivered to the carriers was a function of time of irradiance exposure, W/cm^2^ X time (seconds) of exposure, or J/cm^2^. The UV-C emitters were turned on, allowed to warm up for 10 minutes outside of the test room, and returned to the marked locations. Three emitters positioned side-by-side, each rotating 5°, were used to deliver energy to the carriers at the center of the lamp.^10^^,^^12^ Radiometers were connected, and the International Light Technologies software opened sequentially. For each radiometer, the display units were J/cm^2^. The emitters were turned off only after all 3 emitters had reached each measured dose, with the average maximum cumulative dose for the 3 radiometers calculated and recorded.^10^^,^^12^

**Figure 2:**
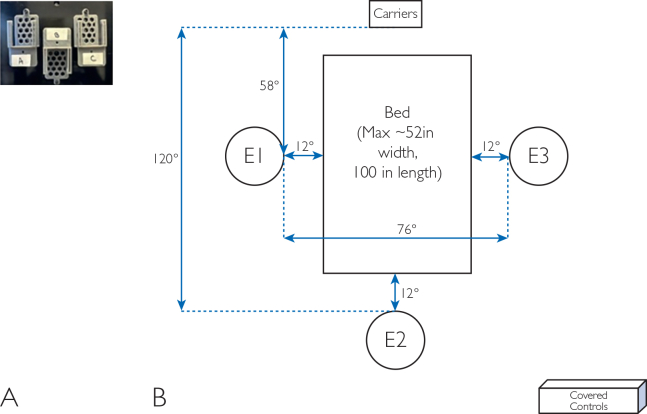
Triangular configuration of emitters each rotating 360°. (A) Pathogen carriers were mounted on an aluminum stand (fixture) at three positions (A=center of stand, B=left of center of stand, and C=right of center of stand) and at a height of 47.5 inches from the floor, center of bulbs. The carriers were positioned on the metal plate (upper left corner of the figure) that was attached to the stand by a screw (hole above position B) that allowed consistency of carrier positioning in relation to the center of the stand while adjusting the height and between experiments. (B) The stand was positioned ten feet from the low-pressure mercury gas UV-C emitter 2 (E2) and 58” from emitter 1 (E1) and emitter 3 (E3). The test carriers and adjacent radiometers were exposed to an increasing dose of UV-C energy. Control carriers were placed inside a cardboard box covered by a foil tray to ensure there was no UVC exposure and placed in the room as far away from emitters as possible. Log reductions (LR) were calculated by comparing the average of treatment samples (final colony-forming units (CFU)) for a given dose to the average of positive controls (initial CFU), log10(initial CFU/final CFU). Following treatment at each target dose, carrier and control slides were removed from the cartridge and processed identically to enumerate CFU. Log reductions were calculated by comparing the average of treatment samples (final CFU) for a given dose to the average of positive controls (initial CFU), log10(initial CFU/final CFU).

Assessment of the impact of delivery of the minimally effective dose through a triangular configuration of emitters: Once the minimally effective dose was established (25 mJ/cm^2^ for *P aeruginosa* [standard carrier] and 650 mJ/cm^2^ for *A flavus* [large carrier]) with the linear emitter configuration, the respective dose was delivered to *P aeruginosa* and *A flavus* carriers positioned on the aluminum stand at positions A, B, and C and at 25.5 and 69.5 in from the floor through a triangular configuration of emitters, one emitter at the head and one on each side of the bed (Figure 2). The delivered dose of irradiation was measured and recorded as described for the linear configuration. As CFU enumeration (below) for each treatment involved 2 dilutions (10^-6^ and 10^-7^), there were a total of 12 CFU enumerations/pathogen (3 positions × 2 dilutions × 2 heights).

### CFU Enumeration:

Linear configuration: Following treatment completion for the entire dose range, the ATCC carrier polycarbonate slides, treatment and control, were removed from their cartridges and each slide placed into a separate 50 mL conical tube (Thermo Fisher Scientific) containing 10 mL of phosphate buffered saline (PBS) (Thermo Fisher Scientific), a 10^-3^ dilution. Conical tubes were vortexed at high speed for 30 seconds with the use of a timer, and 2 serial dilutions were made as follows: 10 μl of solution to 990 μl of PBS and 5 seconds of vortexing on high speed, 10^-6^, and 100 μl of the 10^-6^ solution to 900 μl of PBS, 10^-7^. Each dilution (100 μl) was added to a 5% sheep’s blood agar (Thermo Fisher Scientific) plate from the lowest to highest dilution. The sheep’s blood agar plates were then incubated at 36° C for 24 hours and CFUs quantified. Log reductions were calculated by comparing the average of treatment samples in the 3 positions for each of the 2 dilutions (10^-6^ and 10^-7^ dilutions for each position, n=6) for a given dose to the average of the 3 positive controls for each of the 2 dilutions (n=6) (initial CFU), log_10_(initial CFU/final CFU).^10^^,^^12^ There were 60 LR calculations per pathogen (three positions x 2 dilutions for each irradiation dose (six) × 10 doses per pathogen=60).

Triangular configuration: The above process was used for pathogen carriers exposed to the triangular configuration of emitters. As there were 2 dilutions and 2 heights from the floor for each of the 3 positions on the stand, there were a total of 12 (2 dilutions × 2 heights × 3 positions) CFU enumerations for each pathogen. As above, the impact of the minimally effective dose determined by the linear configuration (25 mJ/cm^2^ for *P aeruginosa* and 650 mJ/cm^2^ for *A flavus*) was assessed.

### Definitions of Pathogen Attenuation

Inactivation was defined by a ≥2-log reduction^17^ when rounded to the whole number. No growth was defined as zero visible CFU following the incubation period.

## Results

For the linear emitter configuration, there were 60 LR calculations for each pathogen. For *P aeruginosa*, the average control CFU was 8.64 × 107. There was no growth (LR≥6) among all treatment samples across the entire dose range, including 25 mJ/cm2. For A flavus, the average control CFU was 1.22 × 108. For standard carriers (1” × 0.9”), inactivation was achieved at 725 mJ/cm2 and maintained throughout the remainder of the dose range, with a mean (SD) LR of 2.08 (0.30) for 725, 1150, 1575, and 2000 mJ/cm2. No growth conditions were not achieved for any dose. For large carriers (1” × 3”), inactivation occurred at 200 mJ/cm2 and was sustained throughout the dose range. No growth conditions were achieved at ≥650 mJ/cm2 (Table).

**Table:** Efficacy of Ultraviolet-C (UV-C) Irradiation Against *Aspergillus flavus* With 10^8^ Inoculum to Standard and Large Polycarbonate Pathogen Carriers

Standard slides 1" × 0.9"	UV-C dose mJ/cm^2^	Mean control CFU/mL	UV-C mean CFU/mL	LR
	50	1.22 × 10^8^	2.8 × 10^7^	0.64
	100	1.22 × 10^8^	1.83 × 10^7^	0.82
	150	1.22 × 10^8^	1.83 × 10^7^	0.82
	200	1.22 × 10^8^	1.43 × 10^7^	0.93
	250	1.22 × 10^8^	1.02 × 10^7^	1.08
	300	1.22 × 10^8^	1.5 × 10^7^	0.91
Inactivation	725	1.22 × 10^8^	1.33 × 10^6^	1.96
	1150	1.22 × 10^8^	5.0 10^5^	2.39
	1575	1.22 × 10^8^	6.67 × 10^5^	2.26
	2000	1.22 × 10^8^	2.33 × 10^6^	1.72
Large Slides 1" × 3"	50	4.52 × 10^7^	1.73 × 10^7^	0.42
	150	4.52 × 10^7^	1.67 × 10^6^	1.43
Inactivation	200	4.52 × 10^7^	8.33 × 10^5^	1.73
	250	4.52 × 10^7^	1.67 × 10^5^	2.4
	350	4.52 × 10^7^	1.67 × 10^5^	2.4
	450	4.52 × 10^7^	0	>6
	500	4.52 × 10^7^	0	>6
	550	4.52 × 10^7^	1.67 × 10^5^	2.4
	650	4.52 × 10^7^	0	>6
	750	4.52 × 10^7^	0	>6

Log reductions were calculated by comparing the average of treatment samples in the 3 positions for each of the 2 dilutions (10^-6^ and 10^-7^ dilutions for each position, n=6) for a given dose to the average of the 3 positive controls for each of the 2 dilutions (n=6) (initial CFU), log_10_(initial CFU/final CFU) [10, 12]. Inactivation was defined by a ≥2-log reduction [17] rounded to the whole number.

Abbreviations: LR, log reduction.

For the triangular emitter configuration, there were 12 LR calculations for each pathogen. For P aeruginosa, the average control CFU was 6.70 × 107. With 25 mJ/cm^2^, there was no growth (LR≥6) among all treatment samples. For A flavus, the average control CFU was 2.45 × 107. With 650 mJ/cm^2^, there was no growth (LR≥6) among all treatment samples.

## Discussion

In this study, we evaluated whether the minimally effective UV-C irradiation dose required for attenuation of a the more pathogenic *S aureus* MLST5 was also effective in attenuating *P aeruginosa*, a Gram-negative pathogen frequently implicated in SSI development^14^^,^^16^ and routinely isolated from the anesthesia machine.^15^ We also evaluated the dose required for inactivation of *A flavus*. We show that 25 mJ/cm^2^ is effective in attenuating *P aeruginosa* contamination of polycarbonate surfaces. For *A flavus*, we found that with >10^8^ inoculum representing high levels of contamination, ≥725 mJ/cm^2^ was required for inactivation and even 2000 mJ/cm^2^ was insufficient to generate no growth conditions. Modeling lower levels of contamination with larger slides, we found that 200 and 650 mJ/cm^2^ were required for inactivation and no growth, respectively. These minimally effective doses (25 mJ/cm^2^ [standard carriers] for *P aeruginosa* and 650 mJ/cm^2^ [larger carriers] for *A flavus*) achieved no growth conditions when delivered by the triangular configuration of UV-C emitters.^10^, ^11^, ^12^, ^13^ As such, 25 mJ/cm^2^ delivered by the triangular configuration will attenuate *P aeruginosa* contamination of polycarbonate surfaces, while high-level surface disinfection prior to UV-C augmentation with ≥650mJ/cm^2^ will be required for *A flavus* inactivation.

Current UV-C treatment strategies are designed to address *S aureus* pathogens tightly associated with SSIs^3^ and the spread of antibiotic resistance between surgical cases on different dates.^28^ It is unknown whether delivery of the minimally effective dose for *S aureus* by an evidence-based, triangular configuration of emitters^10^, ^11^, ^12^, ^13^ requires adjustment to achieve attenuation of *P aeruginosa* or *A flavus*. Adjustment for other pathogens (eg, *Enterococcus*, other Gram-negative spp.) is not indicated for routine disinfection of the anesthesia machine, as such pathogens are rarely associated with anesthesia machine contamination or infections derived from anesthesia workspace reservoirs.^29^^,^^30^

We found that delivery of 25 mJ/cm^2^ through a triangular configuration of UV-C emitters resulted in no growth for *P aeruginosa*, consistent with the impact on *S aureus*, and supporting our primary hypothesis.^10^^,^^11^^,^^13^ While prior work suggests that 99 mJ/cm^2^ is sufficient for inactivation of *A flavus* in water,^20^ we show with testing under high and moderate inoculum conditions (standard and large carrier slides, respectively) that the required dose for *A flavus* inactivation on polycarbonate surfaces is 725 and 200 mJ/cm^2^, for standard and large carriers, respectively. These results are consistent with prior work,^20^^,^^21^ given that inactivation of *A niger* requires ∼180 mJ/cm^2^,^20^ and support the study methodology. High quality surface disinfection (achieving ≤100 CFU)^31^^,^^32^ is needed before UV-C augmentation to reliably achieve *A flavus* inactivation, as the delivery of 650 mJ/cm^2^ was only effective in achieving no growth conditions with use of large carriers that modeled reduced potential for cell clumping. Further work is indicated to determine whether this pathogen is relevant to the anesthesia workspace and to determine the time required for delivery of 650 mJ/cm^2^ to horizontal surfaces with the triangular configuration.^10^, ^11^, ^12^ Until then, the approach described for *Candida auris*^10^*or Clostridioides difficile,*^12^ should be followed.^10^

This study is limited by the lower limit of the dose range, 25 mJ/cm^2^, for *P aeruginosa*. Although a lower dose may also be effective against *P aeruginosa*, the aim of this study was to determine whether the dose required for attenuation of sentinel *S aureus* pathogens^10^^,^^11^ would also be sufficient for more pathogenic and clinically relevant Gram-negative pathogens,^14^, ^15^, ^16^ addressing both in parallel. An additional limitation is that we studied only the treatment of 3 pathogen carriers for each of the doses, a total of 30 carriers assessed at 2 dilutions for 60 total treatment assessments. Although we could have extended the work to additional trials, our finding of no growth with sufficient controls with both the linear and triangular UV-C configurations argued for futility regarding the inclusion of additional treatment evaluations. This is further supported by the reported no growth (≥6-log reduction), where a lesser log reduction (eg, 2 log reduction) is considered a benchmark for disinfection in the health care environment.^17^ Although we assessed only one UV-C technology, the instrument tested has been shown to be more effective than commonly employed, single emitters for delivery of 27 mJ/cm^2^ to horizontal surfaces in a practical time-period.^33^

## Conclusion

In conclusion, the delivery of 25 mJ/cm^2^ of irradiation to polycarbonate surfaces through a triangular UV-C emitter configuration is sufficient for attenuation of *P aeruginosa*. Inactivation of *A flavus* contamination involving polycarbonate surfaces will likely require high-level surface disinfection followed by delivery of ≥650 mJ/cm^2^ of irradiation.

## Potential Competing Interests

Dr Dexter is Director of the Division of Management Consulting of the University of Iowa Department of Anesthesia, which provides consultations to corporations, hospitals, and individuals, including RDB Bioinformatics. He receives no funds personally other than his salary and allowable expense reimbursements from the University of Iowa. He and his family have no financial holdings in any company related to his work. A list of all the Division’s consults is available in his posted curriculum vitae at https://FranklinDexter.net/Contact_Info.htm. Dr Loftus has one or more patents pending and is a partner of RDB Bioinformatics, LLC, at 1055 N 115th St #301 (Omaha, NE, USA), the company that owns OR PathTrac. He receives no funds personally from his involvement in RDB. All other authors report no competing interests.

## Ethics Statement

This experimental design did not include human or animal subjects, tissue, or samples, so it was exempt from approval of the local ethics committee, thus there was no number assigned.
