# Supplementary material for: Assessment of Ultraviolet-C Inactivation of Pseudomonas aeruginosa and Aspergillus flavus in the Anesthesia Workspace
Source: Mayo Clin Proc Innov Qual Outcomes. 2026 Jul 28;10(5):100740. doi: 10.1016/j.mayocpiqo.2026.100740 (PMC13449353; doi:10.1016/j.mayocpiqo.2026.100740)
Supplement: Supplementary file 1 [file mmc1.docx]

**Supplementary Material**

**Additional Information Regarding Pathogen Carrier Preparation:**

This technology facilitates reproducible use of a standard test method (American Society for Testing and Materials (ASTM) 3135: Standard Practice for Determining Antimicrobial Efficacy of Ultraviolet Germicidal Irradiation Against Microorganisms on Carriers with Simulated Soil) for assessment of disinfection efficacy.^22^ Per the manufacturer, the MB-06-10 ‘Standard Operating Procedure for Germicidal Spray Products as Disinfectants’ (GSPT) ^23^ and MB-25-05, Standard Operating Procedure for OECD Quantitative Method for Evaluating Bactericidal and Mycobactericidal Activity of Microbicides Used on Hard, Non-Porous Surfaces^24^ procedures were followed for the preparation of vegetative bacterial cells.^22-24^ Synthetic broth plus the addition of glucose was used where possible rather than nutrient broth or trypticase soy broth (MB-25-05) ^24^ due to the defined composition and improved consistency of synthetic broth as compared to undefined media such as nutrient broth and trypticase soy broth.^24^ Growth conditions were executed according to EPA MB-06-10 ^23^ except for incubation time, which was followed according to MB-25-05.^24^ The 18-24-hour growth period as defined in MB-25-05 ^24^ was leveraged because it is expected to result in consistent growth of cells in the stationary phase rather than longer growth periods that may result in cells entering the death phase of the growth cycle. These procedures are considered to generate consistent preparation of viable microorganisms for antimicrobial testing, including for UV-C.^22-24^

The method for preparation of *P. aeruginosa* inoculum involved synthetic broth with glucose and incubation at 35.0 ± 1°C for 24 ± 1 hr. The method for preparation of *Aspergillus flavus* involved incubation on trypticase soy agar (TSA) at 35.0 ± 1°C for 24 ± 4 hr followed by incubation at 18-22°C for 5-7 days to allow production of conidiospores. Conidiospores were harvested from the agar using a tween 80 solution (0.05%) and purified via use of a 40 µm cell strainer.
